# Higher body mass index is not a protective risk factor for 28-days mortality in critically ill patients with acute kidney injury undergoing continuous renal replacement therapy

**DOI:** 10.1080/0886022X.2019.1650767

**Published:** 2019-08-19

**Authors:** Hai Wang, Yu Shi, Zheng-Hai Bai, Jun-Hua Lv, Jiang-Li Sun, Hong-Hong Pei, Zheng-Liang Zhang

**Affiliations:** Emergency Department & EICU, The Second Affiliated Hospital of Xi’an Jiaotong University, Xi'An, Shaanxi, China

**Keywords:** Body mass index, acute kidney injury, continuous renal replacement therapy, 28-day mortality

## Abstract

**Background:** Acute kidney injury (AKI) requiring continuous renal replacement therapy (CRRT) is a fatal and common clinical disorder in critically ill patients. Recent studies have shown that the relationship between BMI and the outcome of patients with AKI undergoing CRRT is conflicting.

**Methods:** A retrospective cohort study based on data reuse. Univariate analysis, multi-factor regression analysis and subgroup analyses were used to explore the association of the BMI with the 28-days mortality risk in patients with AKI undergoing CRRT.

**Results:** From January 2009 to September 2016, a total of 1120 cases met the inclusion criteria and were enrolled in this study. The univariate analysis showed that BMI was associated with 28-days mortality of patients with AKI undergoing CRRT, its HR value was 0.98 (0.96, 0.99). The multi-factor regression analysis showed that BMI was not associated with 28-days mortality of patients with AKI undergoing CRRT in the four models, the adjusted HR value of four models were 1.00 (0.96, 1.04), 1.01 (0.97, 1.04), 1.00 (0.96, 1.04) and 1.00 (0.96, 1.04), respectively. The subgroups analyses showed that the BMI was a risk factor of the 28-days mortality in patients with AKI undergoing CRRT when GFR ≥30 mL/min, its HR value was 1.04 (1.01, 1.09).

**Conclusion:** Higher BMI was not a protective risk of 28-day mortality in patients with AKI undergoing CRRT. Especially, when GFR ≥30 mL/min, higher BMI increased the risk of the 28-day mortality rate in patients with AKI undergoing CRRT.

## Introduction

Body mass index (BMI) is a simple and useful index for assessing the nutritional status [1]. It has been reported that BMI may be associated with the prognosis of critically ill patients [2]. However, the relationship of BMI with the outcome of critically ill patients is conflicting. In critically ill patients, some studies had shown that obese patients were likely to have higher mortality than others, while other studies had shown that the BMI was not associated with the mortality rate [3,4]. Recent studies had shown that obese patients had lower mortality rates compared to underweight patients [5–7].

Acute kidney injury (AKI) requiring continuous renal replacement therapy (CRRT) is a serious disorder and common in critically ill patients [8]. Additionally, patients with AKI had been found to have worse outcomes which increases the medical costs [9]. In a retrospective single-center study, it was found that BMI was significantly associated with the development of AKI, overweight patients showed higher incidence of AKI and hospital mortality compared to underweight or normal patients [10]. Recently, two studies had shown that high BMI conferred survival benefits to AKI patients undergoing CRRT compared to underweight or normal patients [6,7]. Therefore, researchers have pondered on whether a higher BMI would be a protective risk for the prognosis of patients with AKI undergoing CRRT, and this inspired our interest to explore the relationship of the BMI with mortality in patients with AKI undergoing CRRT.

## Methods

### Study design

A retrospective cohort study of data reuse.

### Objection

To explore the relationship of BMI with the 28-day mortality of patients with AKI undergoing CRRT.

### Data source

Data was provided by Seung Hyeok Han, which was stored in the dryad database (https://datadryad.org//resource/doi:10.5061/dryad.6v0j9) [11]. The database is a public data repository which contains data uploaded by authors to make their research data available for future research.

### Inclusion criteria

Patients with stage 2 AKI according to the Acute Kidney Injury Network (AKIN) criteria; treated with CRRT.

### Exclusion criteria

Age less than 18 years; preexisting CKD or dialysis or CRRT before the study; pregnancy or lactation; postrenal obstruction; prior kidney transplantation; the data that the value of BMI was missing.

### Participants enrollment

From January 2009 to September 2016, 2391 patients undergoing CRRT, of which 1271 patients were excluded due to the following factors: stage I AKIN (*n* = 281), age less than 18 years old (*n* = 42), preexisting CKD or dialysis or CRRT before the study (*n* = 585), pregnancy (*n* = 12), postrenal obstruction (*n* = 263), prior kidney transplantation (*n* = 64), missing value (*n* = 20) and outliers (*n* = 4). Finally, a total of 1120 cases met the inclusion criteria. Patients were categorized into four groups based on Asia criteria of obesity. Asia criterion: underweight (<18.5 kg/m^2^), normal (18.5–22.99 kg/m^2^), overweight (23.0–24.99 kg/m^2^), and obesity (≥25 kg/m^2^) according to BMI classification by the Committee of Clinical Practice Guidelines and Korean Society for the Study of Obesity [12].

### Collection of clinical and biochemical data

Demographic and clinical data including age, sex, BMI (body mass index), SBP (Systolic Blood Pressure), DBP (diastolic blood pressure), CRRT cause and comorbidities were recorded. The following biochemical laboratory data at 0 h such as HB (hemoglobin), WBC (white blood cell), Cr (serum creatinine), phosphate (0 h), ALB (albumin), HCO_3_^–^ (bicarbonate), K^+^ (potassium), BUN (blood urea nitrogen), C-reactive protein (CRP), GFR (glomerular filtration rate). Disease severity index: SOFA score, APACHE II score and CCI score (Charlson comorbidity index).

### CRRT protocol

Nephrologists decided whether or not to initiate CRRT, upon the development of AKI in ICU patients. The indications of CRRT included uncontrolled volume overload, intractable hyperkalemia or metabolic acidosis. The applied model of CRRT was CVVH (continuous veno-venous Hemofiltration) through the internal jugular, subclavian, or femoral vein. CRRT was started at a blood flow rate of 100 mL/min, and up to 150 mL/min. The total effluent volume as a sum of dialysis and replacement dose was targeted to deliver ≥35 mL/kg/h in all patients.

### Statistical analysis

(1) Statistical description: Mean ± standard deviation (x ± s) was used for continuous variables of baseline data in the groups, and counts data were shown by numerical values and percentages. (2) Univariate analysis was carried out to detect the possible risk that may be associated with 28-day mortality. (3) In multi-factor analysis, we adjusted the possible variables that may affect the prognosis of patients to determine the relationship between BMI and 28-day mortality. (4) Sensitivity analysis was carried out for diabetes, hypertension, congestive heart failure, sepsis, GFR, mechanical ventilation, CRRT dose, CRP and SOFA score to further verify the relationship between BMI and 28-day mortality. All statistical analyses were performed by EmpowerStats (version numbers: 2018-05-05, Copyright 2009 X&Y Solutions, Inc) and R software. *p* < 0.05 was considered as a statistical difference.

## Results

### Baseline characteristics

The clinical characteristics and laboratory findings of all patients were shown in Table 1. A total of 1120 cases met the inclusion criteria and were enrolled in this study. The mean age of underweight group, normal group, overweight group and obesity group were 63.10 ± 17.48, 64.85 ± 13.73, 63.94 ± 13.61 and 61.24 ± 14.59 years, *p* = 0.006, respectively. The mean BMI of four groups were 16.70 ± 1.58, 21.04 ± 1.22, 24.00 ± 0.57 and 28.20 ± 3.51 kg/m^2^, *p* < 0.001. The difference of HCO_3_^–^, phosphate (0 h), phosphate (24 h), CRP, GFR, CRRT dose and AKIN score between the four groups were significant, *p* < 0.05. The difference in other clinical characteristics and laboratory findings between the groups were not significant, *p* > 0.05 (see Table 1).

**Table 1.** The clinical characteristics of patients.

Variables	Underweight (*n* = 90)	Normal (*n* = 401)	Overweight (*n* = 224)	Obesity (*n* = 405)	*p* Value
Age, year	63.10 ± 17.48	64.85 ± 13.73	63.94 ± 13.61	61.24 ± 14.59	0.006
Sex (M/F)	53/37	242/159	154/70	238/167	0.082
BMI, kg/m^2^	16.70 ± 1.58	21.04 ± 1.22	24.00 ± 0.57	28.20 ± 3.51	<0.001
Myocardial infarction, *n* (%)	6 (6.67%)	44 (10.97%)	17 (7.59%)	36 (8.89%)	0.400
Congestive heart failure, *n* (%)	18 (20.00%)	67 (16.71%)	40 (17.86%)	54 (13.33%)	0.268
Cerebrovascular disease, *n* (%)	8 (8.89%)	45 (11.34%)	25 (11.16%)	35 (8.64%)	0.566
Diabetes mellitus, *n* (%)	22 (24.72%)	138 (34.41%)	83 (37.05%)	146 (36.05%)	0.187
Hypertension, *n* (%)	38 (42.22%)	216 (53.87%)	113 (50.45%)	224 (55.31%)	0.125
COPD, *n* (%)	11 (12.22%)	34 (8.48%)	14 (6.25%)	21 (5.19%)	0.066
MAP, mmHg	76.98 ± 14.99	77.48 ± 14.29	77.35 ± 14.28	77.71 ± 14.46	0.988
WBC, 10^9^/L	13.56 ± 11.79	13.60 ± 11.60	14.67 ± 12.58	13.93 ± 10.06	0.572
Hb, g/dL	9.56 ± 2.13	9.67 ± 2.10	9.42 ± 2.26	9.69 ± 2.31	0.403
BUN, mg/dL	60.70 ± 35.26	55.83 ± 28.07	56.79 ± 31.36	54.57 ± 29.82	0.634
Cr, mg/dL	1.03 ± 0.49	1.69 ± 6.10	1.12 ± 0.61	1.31 ± 3.59	0.840
K^+^, mmol/L	4.60 ± 1.05	4.62 ± 1.04	4.72 ± 1.13	4.77 ± 1.13	0.389
HCO3-, mmol/L	16.70 ± 5.70	17.68 ± 6.01	16.38 ± 5.75	16.54 ± 5.35	0.023
Phosphate (0 h), mg/dL	5.78 ± 2.90	5.46 ± 2.27	5.86 ± 2.55	5.92 ± 2.27	0.024
Phosphate (24 h), mg/dL	4.43 ± 2.62	4.30 ± 2.23	4.57 ± 2.20	4.74 ± 2.26	0.001
Alb, g/dL	2.48 ± 0.59	2.60 ± 0.58	2.61 ± 0.60	2.65 ± 0.58	0.239
CRP, mg/L	94.63 ± 105.40	122.54 ± 115.61	110.66 ± 105.64	102.6 ± 102.02	0.046
GFR, mL/min	35.22 ± 22.51	32.44 ± 22.51	31.75 ± 22.63	28.86 ± 18.52	0.007
Mechanical ventilation	70 (77.78%)	319 (79.55%)	176 (78.57%)	313 (77.28%)	
APACHE II score	25.81 ± 7.55	27.86 ± 7.47	27.64 ± 8.57	26.77 ± 8.13	0.082
SOFA score	11.44 ± 3.50	11.92 ± 3.66	12.10 ± 3.56	12.44 ± 3.43	0.085
CRRT dose, mL/kg/h	38.38 ± 5.72	37.47 ± 4.16	36.71 ± 4.06	35.51 ± 5.26	<0.001
2 h urine at CRRT initiation, mL	60.68 ± 95.17	64.88 ± 87.58	84.09 ± 113.74	73.75 ± 109.15	0.098
AKIN stages					
2	14 (15.56%)	122 (30.42%)	67 (29.91%)	90 (22.22%)	0.003
3	76 (84.44%)	279 (69.58%)	157 (70.09%)	315 (77.78%)	
CRRT causes					
Volume overload, *n* (%)	10 (11.11%)	53 (13.22%)	32 (14.29%)	57 (14.07%)	0.991
Metabolic acidosis, *n* (%)	22 (24.44%)	80 (19.95%)	47 (20.98%)	88 (21.73%)	
Hyperkalemia, *n* (%)	5 (5.56%)	20 (4.99%)	12 (5.36%)	19 (4.69%)	
Uremia, *n* (%)	10 (11.11%)	42 (10.47%)	18 (8.04%)	43 (10.62%)	
Oliguria, *n* (%)	25 (27.78%)	111 (27.68%)	56 (25.00%)	98 (24.20%)	
Other*, n* (%)	18 (20.00%)	95 (23.69%)	59 (26.34%)	100 (24.69%)	
AKI cause					
Sepsis, *n* (%)	67 (71.28%)	292 (72.82%)	155 (69.20%)	268 (66.17%)	0.269
Nephrotoxin, *n* (%)	2 (2.13%)	11 (2.74%)	8 (3.57%)	16 (3.95%)	
Ischemia, *n* (%)	3 (3.19%)	36 (8.98%)	22 (9.82%)	36 (8.89%)	
Surgery, *n* (%)	7 (7.45%)	27 (6.73%)	16 (7.14%)	43 (10.62%)	
Others, *n* (%)	15 (15.96%)	35 (8.73%)	23 (10.27%)	42 (10.37%)	

### The results of univariate analysis

The univariate analysis showed that BMI, congestive heart failure, diabetes mellitus, hypertension, MAP, phosphate (0 h), phosphate (24 h), mechanical ventilation, Hb, Cr, Alb, 2 h urine output at CRRT initiation, APACHE II score, SOFA score and CRRT causes were associated with the 28-day mortality of patients with AKI undergoing CRRT (see Table 2).

**Table 2.** Univariate analysis.

Variables	Statistics	28-day mortality
Age, year	63.20 ± 14.42	1.00 (1.00, 1.01), 0.788
BMI, kg/m^2^	23.78 ± 4.58	0.98 (0.96, 0.99), 0.024
Sex		
M	705 (61.63%)	1.00 (Reference)
F	439 (38.37%)	0.94 (0.80, 1.09), 0.396
Myocardial infarction, *n* (%)	112 (9.79%)	0.89 (0.70, 1.15), 0.379
Congestive heart failure, *n* (%)	186 (16.26%)	0.77 (0.63, 0.95), 0.016
Cerebrovascular disease, *n* (%)	114 (10.00%)	0.84 (0.65, 1.08), 0.180
Peripheral vascular disease, *n* (%)	46 (4.02%)	0.82 (0.56, 1.20), 0.311
Diabetes mellitus, *n* (%)	398 (34.82%)	0.85 (0.73, 1.00), 0.044
Hypertension, *n* (%)	601 (52.53%)	0.70 (0.60, 0.81), <0.001
COPD, *n* (%)	80 (6.99%)	0.81 (0.60, 1.11), 0.192
MAP, mmHg	77.40 ± 14.62	0.98 (0.98, 0.99), <0.001
K^+^, mmol/L	4.70 ± 1.10	1.02 (0.95, 1.09), 0.573
HCO_3_^−^, mmol/L	16.91 ± 5.72	0.99 (0.97, 1.00), 0.111
Phosphate (0 h), mg/dL	5.75 ± 2.42	1.06 (1.03, 1.09), <0.001
Phosphate (24 h), mg/dL	4.57 ± 2.32	1.16 (1.13, 1.19), <0.001
Mechanical ventilation	898 (78.57%)	1.83 (1.49, 2.24), <0.001
WBC, 10^9^/L	14.13 ± 13.15	1.00 (1.00, 1.00), 0.060
Hb, g/dL	9.63 ± 2.22	0.95 (0.92, 0.98), 0.003
BUN, mg/dL	55.85 ± 29.96	1.00 (1.00, 1.00), 0.135
Cr, mg/dL	2.73 ± 1.62	0.91 (0.87, 0.96), <0.001
Alb, g/dL	2.61 ± 0.58	0.68 (0.60, 0.77), <0.001
CRP, mg/L	110.36 ± 108.05	1.00 (1.00, 1.00), 0.293
GFR, ml/min	31.28 ± 21.17	1.00 (1.00, 1.01), 0.095
CRRT dose, ml/kg/h	36.65 ± 4.80	1.01 (0.99, 1.02), 0.457
2 h urine at CRRT initiation	71.68 ± 102.24	1.00 (1.00, 1.00), <0.001
APACHE II score	27.32 ± 7.97	1.03 (1.02, 1.04), <0.001
SOFA score	12.10 ± 3.55	1.17 (1.14, 1.20), <0.001
AKIN stages		
2	298 (26.05%)	1.00 (Reference)
3	846 (73.95%)	1.03 (0.87, 1.21), 0.768
CRRT causes		
Volume overload, *n* (%)	160 (13.99%)	1.00 (Reference)
Metabolic acidosis, *n* (%)	242 (21.15%)	1.35 (1.04, 1.74), 0.023
Hyperkalemia, *n* (%)	58 (5.07%)	1.50 (1.03, 2.18), 0.033
Uremia, *n* (%)	115 (10.05%)	0.90 (0.66, 1.25), 0.538
Oliguria, *n* (%)	294 (25.70%)	1.02 (0.79, 1.32), 0.862
Other, *n* (%)	275 (24.04%)	1.26 (0.98, 1.63), 0.072
AKI causes		
Sepsis, *n* (%)	798 (69.76%)	1.00 (Reference)
Nephrotoxin, *n* (%)	37 (3.23%)	0.93 (0.61, 1.41), 0.723
Ischemia, *n* (%)	98 (8.57%)	1.06 (0.81, 1.38), 0.667
Surgery, *n* (%)	94 (8.22%)	0.83 (0.62, 1.11), 0.200
Others, *n* (%)	117 (10.23%)	1.34 (1.05, 1.70), 0.018

### The results of multi-factor regression analysis

In the multi-factor regression analysis, we found that when BMI was used as a continuous variable, it was not associated with the 28-day mortality of patients with AKI undergoing CRRT. When BMI was employed as a continuous variable, the adjusted HR value in the four models were separately 1.00 (0.96, 1.04), 1.01 (0.97, 1.04), 1.00 (0.96, 1.04) and 1.00 (0.96, 1.04). When BMI was applied as a classification variable, it was also not associated with the 28-day mortality of patients with AKI undergoing CRRT in the four models (see Table 3).

**Table 3.** Multi-factor cox regression analysis for 28-day mortality.

Exposure	Non-adjusted HR, *p* Value	Adjusted HR, *p* Value
*Model 1*		
BMI	0.99 (0.96, 1.03), 0.612	1.00 (0.96, 1.04), 0.987
BMI		
18.5–22.99, kg/m^2^	1.00 (Reference)	1.00 (Reference)
<18.5, kg/m^2^	1.21 (0.88, 1.67), 0.248	1.20 (0.87, 1.66), 0.265
23–24.99, kg/m^2^	1.05 (0.83, 1.32), 0.710	1.00 (0.79, 1.27), 0.983
≥25, kg/m^2^	1.00 (0.74, 1.37), 0.981	0.96 (0.71, 1.31), 0.806
*Model 2*		
BMI	0.99 (0.96, 1.03), 0.612	1.01 (0.97, 1.04), 0.719
BMI		
18.5–22.99, kg/m^2^	1.00 (Reference)	1.00 (Reference)
<18.5, kg/m^2^	1.21 (0.88, 1.67), 0.248	0.90 (0.57, 1.42), 0.648
23–24.99, kg/m^2^	1.05 (0.83, 1.32), 0.710	1.10 (0.80, 1.52), 0.547
≥25, kg/m^2^	1.00 (0.74, 1.37), 0.981	1.21 (0.84, 1.74), 0.312
*Model 3*		
BMI	0.99 (0.96, 1.03), 0.612	1.00 (0.96, 1.04), 0.960
BMI		
18.5–22.99, kg/m^2^	1.00 (Reference)	1.00 (Reference)
<18.5, kg/m^2^	1.21 (0.88, 1.67), 0.248	0.87 (0.55, 1.37), 0.543
23–24.99, kg/m^2^	1.05 (0.83, 1.32), 0.710	1.18 (0.85, 1.64), 0.313
≥25, kg/m^2^	1.00 (0.74, 1.37), 0.981	1.26 (0.87, 1.82), 0.221
*Model 4*		
BMI	0.99 (0.96, 1.03), 0.612	1.00 (0.96, 1.04), 0.931
BMI		
18.5–22.99, kg/m^2^	1.00 (Reference)	1.00 (Reference)
<18.5, kg/m^2^	1.21 (0.88, 1.67), 0.248	1.01 (0.64, 1.61), 0.959
23–24.99, kg/m^2^	1.05 (0.83, 1.32), 0.710	1.19 (0.85, 1.66), 0.303
≥25, kg/m^2^	1.00 (0.74, 1.37), 0.981	1.17 (0.81, 1.70) ,0.402

Model 1: Adjusted for age, sex, diabetes mellitus, hypertension, COPD, myocardial infarction, congestive heart failure, cerebrovascular disease, AKI cause and CRRT cause.

Model 2: Model 1 and WBC, Alb, CRP, GFR, Hb, BUN, K^+^, HCO3-, Phosphate (0 h) and Phosphate (24 h).

Model 3: Model 2 and Mechanical ventilation at CRRT initiation, CRRT dose, 2 h urine output at CRRT initiation.

Model 4: Model 3 and APACHE II score, SOFA score.

### The results of subgroup analysis of multi-factor regression analysis

The sensitivity analysis was carried out for diabetes, hypertension, congestive heart failure, sepsis, mechanical ventilation, CRRT dose, CRP and SOFA score, which revealed that a higher BMI did not reduce the risk of the 28-day mortality rate in critically ill patients. The subgroup analyses showed that the BMI was associated with the risk of 28-days mortality in patients with AKI undergoing CRRT when GFR ≥30 mL/min, its HR value was 1.04 (1.01, 1.09) (see Table 4).

**Table 4.** Subgroup analysis of multi-factor cox regression analysis for 28-day mortality.

Subgroup variables	Non-adjusted HR, *p* Value	Adjusted HR, *p* Value
Diabetes		
YES	0.99 (0.96, 1.02), 0.384	0.99 (0.95, 1.02), 0.498
NO	0.99 (0.96, 1.01), 0.236	1.04 (1.00, 1.08), 0.056
Hypertension		
YES	0.98 (0.96, 1.01), 0.153	1.01 (0.98, 1.04), 0.614
NO	0.99 (0.97, 1.02), 0.680	1.04 (0.99, 1.09), 0.087
Congestive heart failure		
YES	1.00 (0.96, 1.05), 0.829	1.03 (0.98, 1.09), 0.231
NO	0.98 (0.96, 1.00), 0.043	1.00 (0.97, 1.03), 0.885
Sepsis		
YES	1.00 (0.97, 1.03), 0.806	0.98 (0.94, 1.02), 0.321
NO	0.98 (0.96, 1.00), 0.0517	1.02 (0.99, 1.05), 0.271
GFR		
GFR < 30	0.98 (0.96, 1.01), 0.144	0.98 (0.95, 1.02), 0.365
GFR ≥ 30	0.99 (0.97, 1.02), 0.715	1.04 (1.01, 1.09), 0.049
Mechanical ventilation		
YES	0.97 (0.96, 0.99), 0.012	1.01 (0.98, 1.03), 0.653
NO	1.03 (0.98, 1.07), 0.276	1.02 (0.94, 1.11), 0.565
CRRT dose		
CRRT dose < 35	1.01 (0.98, 1.04), 0.400	0.99 (0.95, 1.03), 0.701
CRRT dose ≥ 35	0.97 (0.95, 0.99) 0.007	1.03 (1.00, 1.07), 0.077
CRP		
CRP < 50	1.00 (0.97, 1.03), 0.857	1.02 (0.99, 1.05), 0.254
50 ≤ CPR < 100	0.98 (0.93, 1.03), 0.396	1.00 (0.93, 1.08), 0.903
CRP ≥ 100	0.98 (0.95, 1.01), 0.183	0.99 (0.95, 1.03), 0.608
SOFA score		
SOFA score < 12	1.00 (0.97, 1.03), 0.889	1.02 (0.98, 1.07), 0.344
SOFA score ≥ 12	0.97 (0.95, 0.99), 0.004	1.01 (0.98, 1.04), 0.652

Adjusted variables (without the subgroup analysis variables themselves): age, sex, diabetes mellitus, hypertension, myocardial infarction, cerebrovascular disease, COPD, congestive heart failure, CRRT cause, AKI cause, CRRT dose, WBC, Alb, Hb, CRP, GFR, BUN, K+, HCO_3_^–^, Phosphate (0 h), Phosphate (24 h), 2 h urine at CRRT initiation, mechanical ventilation at CRRT initiation, APACHE II score and SOFA score.

## Discussion

The multiple factor regression analysis showed that BMI did not decrease the risk of 28-day mortality in critically ill patients with AKI undergoing CRRT. Further sensitivity analyses showed that when GFR ≥30 mL/min, a higher BMI increased the risk of the 28-day mortality in patients with AKI undergoing CRRT.

A prospective study including 82 severely obese patients (mean BMI, 42 ± 6 kg/m^2^) and 124 nonobese patients (mean BMI, 24 ± 4 kg/m^2^) with mechanical ventilation in ICU showed that obesity was not associated either with increased ICU mortality or with hospital mortality [4]. In this study, the researchers controlled the potential confounding factors using multiple factor logistic regression analysis and matched the following variables: age, gender, and the simplified acute physiology (SAPS) II score between obese patients and nonobese patients. In another study which included 4698 patients mechanically ventilated, it was found that being overweight was not associated with high mortality in ICU patients after adjusting for the following variables: age, sex, SAPS II, body mass index category and type of ventilation [3]. In our study, we also found that obesity was not associated with the 28-day mortality of patients with AKI undergoing CRRT.

A cohort study that was conducted for over 2 years in six medical-surgical ICUs that enrolled 1698 patients showed that BMI below 18.5 kg/m^2^ was independently associated with high mortality (adjusted OR: 1.63; 95% confidence intervals 1.11–2.39). The BMI >30 kg/m^2^ reduced the risk of mortality (adjusted OR: 0.60, 95% confidence intervals 0.40–0.88) and BMI between (18.5–24.9 kg/m^2^) and (25–29.9 kg/m^2^) were not associated with high mortality [5]. The conclusion of this study was inconsistent with that of our study. There are several possible reasons: (1) the study populations were different, our study only included ICU patients with AKI undergoing CRRT, while this study included all the ICU patient; (2) the outcome indicators were not similar, the outcome in our study was 28-day mortality, while the outcomes in this study were ICU and hospital mortality; (3) we adjusted for the possible confounding factors to determine the independent effect of BMI on the 28-day mortality, but the possible confounding factors were not adjusted for in this study.

Another observational study including 212 patients with AKI undergone CRRT found that a higher BMI was beneficial to AKI patients unlike a low BMI value [6]. In study by Seung Hyeok Han published in 2018, they also found that a survival benefit of high BMI in AKI patients (SOFA score ≥ 12) undergoing CRRT [7]. The above two studies were performed by Seung Hyeok Han. However, our conclusions were inconsistent with theirs. This may be due to the following reasons: (1) At the beginning of statistical analysis, all cases with missing BMI data and outliers were excluded from the study; (2) The first study by Seung Hyeok Han enrolled 212 patients with AKI undergone CRRT, which was significantly smaller compared to our study, thus the conclusion of our study was more reliable. (3) The second study by Seung Hyeok Han adjusted the following variables: age, sex, CCI score, septic AKI, MAP, eGFR, SOFA score, WBC, Alb and CRRT dose, but did not adjust for the following variables: myocardial infarction, diabetes, congestive heart failure and hypertension, CRP, AKI cause, mechanical ventilation and phosphate which had been reported to occur in critically ill patients [13–17]. (4) We tested the collinearity of the variables included in the statistical analysis, and found that VIF of all variables was less than 2, hence there was no statistical collinearity in the included variables. Nevertheless, further analysis of the included variables showed that SOFA scores comprised of MAP, CR and AKIN stage, and that age and complications exhibited collinearity with CCI scores. Therefore, to avoid the instability of the model caused by collinearity among variables, we did not include MAP, Cr, AKIN stage and CCI in the statistical analysis. The collinearity between variables was not taken into account in the study by Seung Hyeok Han, which was likely to affect the reliability of their results. Therefore, our conclusion was more reliable compared to the above studies.

There several studies that had shown that the following factors: age, platelet count, APACHE II score, serum creatinine level, a urine output of <0.05 mL/kg/h the first day, eGFR <45 mL/min, et al were associated with the prognosis of patients with AKI undergoing CRRT [18,19]. Moreover, there was study that reported that BMI was a risk for AKI, but was not associated with prognosis of the patients with sepsis treated with CRRT [20].

In our subgroup analysis, the BMI was found to be a risk factor for the 28-days mortality in patients with AKI undergoing CRRT when GFR ≥30 mL/min. A meta-analysis including 39 general population cohorts (*n* = 5 459 014) revealed that higher BMI was an independent risk factor for GFR decline and death in patients who have normal or reduced levels of estimated GFR [21]. This may explain why the BMI was a risk factor for the 28-days mortality in patients with AKI undergoing CRRT when GFR ≥30 mL/min. Even so, we found that the BMI was not associated with 28-days mortality of patients with AKI undergoing CRRT when GFR <30 mL/min. A possible rationale was that when GFR <30 mL/min, the renal function was impaired, which affected the prognosis.

### Strength of the study

This study present new findings that BMI was not associated with the 28-day mortality in patients with AKI undergoing CRRT. Here, we controlled more possible confounding factors than previous studies which avoided obvious statistical errors, making our conclusions more reliable.

### Limitations of the study

Only patients with AKI and undergoing CRRT were enrolled, which limited the application of our conclusions. There was no data on other risk factors of mortality such as other interventions, cardiac support during the ICU/hospital stay etc. which might influence our conclusions.

## Conclusion

Higher BMI was not a protective risk of 28-day mortality in patients with AKI undergoing CRRT. Especially, when GFR ≥30 mL/min, higher BMI increased the risk of the 28-day mortality rate in patients with AKI undergoing CRRT.
